# Training and capacity development in patient-oriented research: Ontario SPOR SUPPORT Unit (OSSU) initiatives

**DOI:** 10.1186/s40900-023-00415-8

**Published:** 2023-02-25

**Authors:** Colin MacArthur, Rob Van Hoorn, John Lavis, Sharon Straus, Nicola Jones, Lorraine Bayliss, Amanda L. Terry, Susan Law, Charles Victor, Denis Prud’homme, John Riley, Moira Stewart

**Affiliations:** 1 Peter Gilgan Centre for Research and Learning, Hospital for Sick Children Research Institute, 686 Bay Street, Toronto, ON M5G 0A4 Canada; 2 Schulich School of Medicine and Dentistry, London, ON Canada; 3 McMaster Health Forum, McMaster University, Hamilton, ON Canada; 4 Keenan Research Center, Li Ka Shing Knowledge Institute, Toronto, ON Canada; 5 Patient Partner, Ontario SPOR SUPPORT Unit, Toronto, ON Canada; 6 Trillium Health Partners, Institute for Better Health, Mississauga, ON Canada; 7 Institute for Clinical Evaluative Sciences, Toronto, ON Canada; 8 University of Moncton, Moncton, NB Canada

**Keywords:** Patient engagement, Capacity building, Patient-oriented research

## Abstract

**Background:**

In Canada, the Canadian Institutes of Health Research launched the Strategy for Patient-Oriented Research (SPOR) in 2011. The strategy defines ‘patient-oriented research’ as a continuum of research that engages patients as partners, focuses on patient priorities, and leads to improved patient outcomes. The overarching term ‘patient’ is inclusive of individuals with personal experience of a health issue as well as informal caregivers including family and friends. The vision for the strategy is improved patient experiences and outcomes through the integration of patient-oriented research findings into practice, policy, and health system improvement. Building capacity in patient-oriented research among all relevant stakeholders, namely patients, practitioners, organizational leaders, policymakers, researchers, and research funders is a core element of the strategy.

**Main body:**

The objective of this paper is to describe capacity building initiatives in patient-oriented research led by the Ontario SPOR SUPPORT Unit in Ontario, Canada over the period 2014–2020.

**Conclusion:**

The Ontario SPOR SUPPORT Unit Working Group in Training and Capacity Development has led numerous capacity building initiatives: developed a Capacity Building Compendium (accessed greater than 45,000 times); hosted Masterclasses that have trained hundreds of stakeholders (patients, practitioners, organizational leaders, policymakers, researchers, and trainees) in the conduct and use of patient-oriented research; funded the development of online curricula on patient-oriented research that have reached thousands of stakeholders; developed a patient engagement resource center that has been accessed by tens of thousands of stakeholders; identified core competencies for research teams and research environments to ensure authentic and meaningful patient partnerships in health research; and shared these resources and learnings with stakeholders across Canada, North America, and internationally.

## Plain English summary

In 2011, Canada developed a Strategy for Patient-Oriented Research. The aim of the strategy was to ensure that patients were included as equal partners in research, with the goal to improve the patient experience and enhance health outcomes using research findings to influence clinical care, policy, and health system improvement. Building capacity in patient-oriented research is a core element of the strategy. Since 2014, the Ontario SPOR SUPPORT Unit has led numerous initiatives to build capacity in patient-oriented research. Successes include a Capacity Building Compendium (a catalogue of resources that has been accessed greater than 45,000 times); courses on how to do and how to use patient-oriented research that have trained hundreds of patients, practitioners, organizational leaders, policymakers, and researchers; created online patient-oriented research materials; developed a patient engagement resource center; identified what is required to ensure authentic and meaningful patient partnerships in research; and shared these resources and learnings widely.

## Background

Patient and public involvement in health research is an international movement. For example, the National Institute for Health Research in the UK has promoted and supported patient and public involvement in health and social care research over the last 3 decades through organizations such as INVOLVE and the Centre for Engagement and Dissemination [1]. In the United States, the Patient-Centered Outcomes Research Institute (PCORI)—established through an Act of Congress in 2010—has funded hundreds of comparative effectiveness research projects guided by patient values, preferences, and needs [2].

In Canada, the Canadian Institutes of Health Research (CIHR) launched the Strategy for Patient-Oriented Research (SPOR) in 2011 [3]. The strategy defines ‘patient-oriented research’ as a continuum of research that engages patients as partners, focuses on patient priorities, and leads to improved patient outcomes. The overarching term ‘patient’ is inclusive of individuals with personal experience of a health issue as well as informal caregivers including family and friends. The vision for SPOR is improved patient experiences and outcomes through the integration of patient-oriented research findings into practice, policy, and health system improvement [3].

The objective of this paper is to set the context for capacity building within a national SPOR, outline the role of the provincial Ontario SPOR SUPPORT Unit (OSSU), and describe specific capacity building initiatives in patient-oriented research led by OSSU over the period 2014–2020. Where available, for each specific OSSU initiative in capacity building, information will be provided on the rationale for the initiative (i.e., gap addressed by the initiative), learning objectives, patient engagement, stakeholder involvement, outcomes, and evaluations.

## Building capacity in patient-oriented research within the national SPOR

Core elements of SPOR include (a) patient engagement (empowering patients as equal partners in the research process), (b) building capacity in patient-oriented research, (c) innovative clinical trials, (d) multi-disciplinary methodological services and supports, and (e) national research networks. Of note, while capacity development is a stand-alone core element of SPOR, building capacity in patient-oriented research is also mandated across all SPOR core elements.

SPOR aims to build capacity in patient-oriented research among all relevant stakeholders, namely patients, practitioners, organizational leaders, policymakers, researchers, and research funders. The intent is for all stakeholders to be trained, aligned, prepared, and working collaboratively together to support and promote patient-oriented research and to integrate such research findings into practice, policy, and the health system to improve the patient experience and health outcomes at the individual and population level.

In this context, a SPOR Capacity Development Framework has been developed. The framework articulates guiding principles for capacity development in patient-oriented research: (a) ensuring capacity for meaningful patient engagement, (b) mobilizing existing expertise, (c) supporting careers, (d) collaboration, and (e) building capacity to apply research evidence [4]. Given the diverse set of stakeholders, the framework recognizes that all stakeholders in patient-oriented research should receive appropriate training and support, and that access to training, tools, and resources should be equitable.

## The Ontario SPOR SUPPORT Unit (OSSU)

CIHR SPOR funding seeded the development of SPOR SUPPORT (Support for People and Patient-Oriented Research and Trials) Units across all provinces and territories in Canada. The Ontario SPOR SUPPORT Unit (OSSU) was established in 2014 through base funding from CIHR SPOR along with matching funding from the Ontario provincial government. OSSU consists of a network of 15 health research centers in Ontario, supported by a coordinating center.

High-level aims of OSSU are to raise awareness of the importance of the patient perspective in research and to provide infrastructure and process support for patient-oriented research. OSSU principles to enhance patient-oriented research include multiple stakeholder involvement in initiatives, a provincial reach across all of Ontario’s regions and, most importantly highlighting the primacy of the patient voice.

### Patient engagement in OSSU

Patient partners are engaged and involved at all levels of OSSU. For example, at the governance level, patient partners are members of the OSSU Board of Directors and the Operational Management Team. Patient partners lead an OSSU Patient Partners Working Group, one of four working groups providing expertise in core function areas of the SUPPORT Unit. Patient partners are also members of the other OSSU core working groups: Training and Capacity Development, Data Platforms, and Learning Health Systems. Last, OSSU initiatives at the program level, for example, the online training programs in POR, are mandated to have equal and authentic patient partner representation.

OSSU has funded patient-oriented research demonstration projects (IMPACT Awards) and patient-oriented research knowledge translation projects (EMPOWER awards). Both funding opportunities mandated that patients be included as research partners on funding applications. Submissions were also reviewed and ranked by patient partners. These partnerships between patients and researchers in the OSSU funded initiatives have had an impact on clinical care, health policy, and health systems across a range of key health conditions such as mental health, obesity, trauma, complex care, and indigenous health [5, 6].

## Specific OSSU capacity development initiatives

### The OSSU Working Group for Training and Capacity Development

Striking a Working Group for Training and Capacity Development (WGTCD)—to meet a core mandate of SPOR—was an early OSSU capacity-building initiative. The WGTCD membership was selected to ensure a broad representation of stakeholders including patients, providers, policymakers, and researchers, selected from all regions of Ontario. Of note, the published literature has identified the lack of stakeholder training and support, including the lack of tools and resources, as significant barriers to effective patient-oriented research [7–11]. Given these barriers, the aims of the OSSU Working Group on Training and Capacity Development were to build capacity in the *conduct* of patient-oriented research as well as in the *use* of patient-oriented research by patients, practitioners, organizational leaders, policymakers, and researchers.

The WGTCD began by articulating principles to guide its deliberations (see Table 1). Next, the WGTCD conducted an environmental scan of existing patient-oriented research capacity-building initiatives in Ontario to identify gaps in knowledge, attitudes, and skills related to patient-oriented research (see below). The findings of the environmental scan guided the work of the WGTCD in the development of new OSSU capacity-building programs and initiatives to bridge the gaps identified.

**Table 1:** The Ontario SPOR SUPPORT Unit WGTCD guiding principles

1. Include the full range of stakeholders in all initiatives—patients and families, health care professionals, policy makers, researchers, and trainees—with all stakeholders considered equal and where possible, bridge disciplines and sectors
2. Use the patient experience, data, and evidence in an iterative process to identify gaps, select pedagogical approaches, implement activities, conduct formative and summative evaluations, and revise as needed the gaps, activities, and evaluations
3. Identify and build on existing investments in patient-oriented research
4. Plan for sustainability
5. Plan initiatives that are scalable
6. Use transparent decision-making processes
7. Measure outcomes using a variety of evidence-based and experience-based metrics
8. Supplement group-based activities with mentoring and coaching
9. Always advance the public interest
10. Undertake capacity development activities where feasible in French and English

### The OSSU environmental scan, survey, and compendium

Environmental scans of 2013, 2015, and 2017 aimed to catalogue and describe patient-oriented research (POR) capacity building initiatives in Ontario for patients, practitioners, policymakers, managers, and researchers as well as to provide a foundation to inform the development of new and innovative evidence-based capacity building initiatives. The resulting compendiums were designed to be useful to at least two groups: prospective participants looking for POR training; and those intending to develop training programs in POR and looking for models to emulate.

The scans were conducted using online questionnaires sent to the 14 members of the OSSU Operational Management Team (comprised of leaders of research centres in Ontario). Using a snowball sampling technique, the questionnaire was then circulated on by OSSU OMT members to research and policy leaders known to conduct POR training programs. The topics covered by the questionnaire were: availability of the programs; topic covered by the training program, e.g., research methods or knowledge translation and exchange; mode of delivery of the training program such as web-based or face-to-face workshop; and the audience(s) targeted by the training program such as patients, researchers, or other stakeholders.

In 2013, the WGTCD catalogued 28 existing resources, training programs, and courses in patient-oriented research within OSSU research centres and SPOR networks, that constituted the first edition of the OSSU Capacity Building Compendium.

The environmental scan identified strengths and gaps in POR capacity building in Ontario related to target audiences and content. For example, the 2013 survey identified a lack of capacity building initiatives for the patient population to allow meaningful and authentic participation of patients in research.

A third edition of the Compendium was created in 2017 and included 53 entries that represented diverse capacity building content, including free online resources and tools, seminars and symposia series, and training programs. Of these programs, 43% provided training for patients and/or addressed patient/public engagement. The 2017 edition saw a 15% increase in patient/public engagement programs from the earlier editions of the compendium. As of February 2022, the compendium link on the OSSU website at https://ossu.ca/for-researchers/training/ has had 45,000 views.

Gaps identified by the environmental scan, led to new OSSU capacity building initiatives. One was the Masterclass, an in-depth face-to-face (in-person or video linked) training for selected stakeholders on the conduct and use of patient-oriented research. The second was a WGTCD funding competition to support the development of on-line training programs to build capacity in POR that would reach a broader stakeholder group than could be reached by the Masterclass initiative.

### OSSU masterclass in the conduct and use of patient oriented research

The learning objectives of the Masterclass were (i) to develop the competencies needed to conduct and use patient-oriented research in Ontario, (ii) to become familiar with the additional training and other supports available in Ontario to conduct and use patient-oriented research, and (iii) to identify ways that organizations (and the organizations with whom they worked) could better support the conduct and use of patient-oriented research, and how to monitor and evaluate such efforts.

Training was focused both on the conduct of patient-oriented research, i.e., for those conducting original research, and on the use of patient-oriented research, i.e., for those using existing patient-oriented research evidence in decision-making, whether government policymakers, organizational leaders, providers, or patients and families. Evaluation of Masterclasses involved pre-workshop assessments, and post-workshop evaluations.

Intensive three-day Masterclasses for a mix of patients, providers, organizational leaders, policymakers, researchers, and research trainees were held in April 2016, October 2016 and October 2017. Trainers included all stakeholder groups including patients. A condensed version of the Masterclass was provided to government stakeholders who had requested training to enhance the application of patient-oriented research to their policy decision-making processes.

Several criteria were used to select participants for the Masterclass workshops to ensure that each cohort was a balanced group, including role (patient, provider, organizational leader, policymaker, researcher, or research trainee), geographic location within Ontario, equity, diversity, and inclusion criteria, gender, career stage, and experience with patient-oriented research.

The workshops have produced a cadre of advanced learners knowledgeable in POR: 32 patients; 35 health care providers; 34 policy makers; and 43 researchers. The Masterclass offered opportunities to work in groups that were homogenous (patient partners together) or heterogenous (participants drawn from all four groups). The course materials used to help stakeholders develop the necessary competencies to conduct and use patient-oriented research can be found here: https://ossu.ca/resources/master-class/.

The OSSU Masterclass transitioned from in-person sessions to a virtual setting with four Masterclasses running in Winter, Summer and Fall of 2019, and Winter 2020. These virtual sessions trained an additional 73 stakeholders including policymakers, patients, providers, organizational leaders, and researchers. No further virtual sessions were offered after 2020.

### The OSSU online training programs initiative: the patient-oriented research capacity building initiatives awards

The WGTCD administered a Patient-Oriented Research Capacity Building Initiatives Awards competition in 2016. Following a call for applications, a workshop was held with patients and other stakeholders to bring program applicants together to share their proposals and receive feedback. In total, the WGTCD competition funded seven projects that provide online training in POR, all developed with a patient partner co-lead.

As of March 2020, the online POR training programs funded by this initiative (many launching in 2019) have reached greater than 1690 stakeholders—patients, practitioners, organizational leaders, policymakers, and researchers—an average of 240 participants per program, with patients constituting more than half of all participants (71%, greater than 1200 participants). The training programs have been designed to support different communities such as primary care, paediatrics, Francophones, and Indigenous communities. See Table 2 for a listing of the on-line training programs and their learning objectives.

**Table 2:** OSSU patient-oriented research online training programs

Title of project	Program/learning objectives
PORTL-PHC	Self-directed, open access online training resource (4 1-h modules) to build capacity among patients, clinicians, policy makers, and researchers in the conduct and use of patient-oriented research in primary health care
Building capacity in POR to strengthen ontario health system	Online series of courses and webinars on ‘evidence,’ the use of POR, and how the Ontario Health Care System works, all targeted towards citizens
Experiences of patient-researcher partnerships	A module on patient-researcher partnerships composed of video vignettes of patient experiences across different aspects of research partnership to help support patients, caregivers, and researchers interested in learning about working collaboratively as partners on research projects
Indigenous community research partnerships	Online tutorial on the use of a collaborative framework for community-research partnerships designed to assist researchers who are new to research partnerships with First Nations, Inuit, and Métis (FNIM) communities
Patient-oriented research curriculum in child health (PORCCH)	Self-directed, open access online training resource (4 thirty-minute modules) to build capacity among patients/families and researchers in the conduct of patient-oriented research in child health
L’approche de recherche collaborative (ARC) axée sur les patients (collaborative research approach for patient-oriented research)	French-language, online training programs on a collaborative research approach for patient-oriented research developed for patients and caregivers, decision makers and managers, and researchers
Partners in research	A 2-month online course (bi-weekly real time webinars, quizzes, problem-based learning, and group coaching) for patients and researchers to learn how to conduct and engage in patient-oriented research

The OSSU online training programs have had national uptake by SPOR SUPPORT Units across Canada and have also reached international stakeholders. For example, as of September 2022, the patient-oriented research curriculum in child health (PORCCH) has had more than 100,000 unique visitors to the site (www.porcch.ca) from more than 16 countries around the world, and the curriculum has been integrated into hospital family advisory committee onboarding programs as well as international research networks [12]. Taken together, the portfolio of online POR capacity building programs resulting from the WGTCD competition offers something for learners from all backgrounds, disciplines, and experience levels. Formal evaluations of several of these OSSU online training programs have been published [13–15].

### The OSSU patient-engagement resource centre (PERC)

The Patient Engagement Resource Centre (PERC), funded by OSSU, can be found at https://www.patientengagement-phcresearch.com/. PERC has a robust set of processes and committees, including a Patient Committee, that review and continuously update resources and has increased the number and quality of the resources offered (guides, research articles, practical advice, consultations, etc.). The PERC website was accessed 1685 times in 2021, with toolkits and evaluation resources accessed 40% of the time. PERC has a strong Patient Committee that provides feedback, advice and works with OSSU-affiliated programs. The PERC Patient Committee has held two workshops and attended the meetings of several other organizations to disseminate their work, help create new resources, and identify priority areas for further work.

### Core competencies for patient-oriented research initiative

The SPOR Capacity Development Framework was led by an external advisory committee and developed with substantial stakeholder input as well as two large workshops over the period 2013–2015. The WGTCD specifically contributed to the SPOR SUPPORT Unit Council *Core Competencies for Patient-Oriented Research* initiative in 2015. As part of this work, the WGTCD developed an outline of capacities required of patients, practitioners, organizational leaders, policymakers, and researchers for the successful conduct and use of POR. The capacities were identified through an iterative expert consultation process among the members of the WGTCD and members from Patients Canada.

From this work, and other stakeholder input, the SPOR SUPPORT Unit Council identified seven core competencies—effective communication, collaboration, knowledge of the health system, knowledge of health research, understanding of patient-oriented research, awareness of the evolution of patient roles, and the added value of patient involvement in research—that were considered important and conducive to meaningful and authentic patient-oriented research. This work became Appendix 2 of the SPOR Capacity Development Framework [4].

### OSSU capacity-building national collaborations

As per its mandate, the OSSU WGTCD has provided training and capacity building in POR to a variety of stakeholders in a variety of POR settings to increase the cadre and quality of POR practitioners across Ontario. The WGTCD has also collaborated with national training programs such as TUTOR-PHC, an interdisciplinary primary health care research training program, and the recently created SPOR National Training Entity. In addition, the WGTCD has collaborated with national research networks such as the pan-Canadian SPOR Network in Primary and Integrated Health Care Innovations. In summary, the WGTCD has engaged or consulted with SPOR-funded groups (SUPPORT Units, Research Networks, and Training Networks) across Canada from Newfoundland to British Columbia.

### Stakeholder feedback

Stakeholder feedback across all the capacity building initiatives has suggested that the most successful components were those involving face-to-face multi-stakeholder group learning sessions, for example, the heterogeneous group sessions in the Masterclass. These sessions led to a broader appreciation and understanding of different stakeholder perspectives on patient-oriented research, as well as a deeper understanding of the health care system by stakeholders. Less transformative, but still important for information exchange, was the compendium.

### Future directions

In October 2021, the OSSU WGTCD and the OSSU Patient Partner Working Group partnered to host a priority setting event for training and capacity development in POR. Participants included patients, practitioners, researchers, managers, and policymakers. The workshop resulted in the OSSU *Capacity Building Blueprint*, and the report identified eight priority areas for capacity development in phase 2 of SPOR: Equity, Diversity, and Inclusion (EDI); sustainability of successful SPOR phase 1 capacity building initiatives; POR relevance and resilience in emergent health crises; building POR capacity among funding agencies; clarifying patient partner compensation; enhancing mentoring; health services research; and capacity development in Learning Health Systems.

## Conclusion

In conclusion, CIHR SPOR funding has supported numerous OSSU capacity building initiatives in patient-oriented research. The OSSU WGTCD has developed a Capacity Building Compendium (accessed greater than 45,000 times); hosted a number of Masterclasses that have trained hundreds of stakeholders (patients, practitioners, organizational leaders, policymakers, researchers, and trainees) in the conduct and use of patient-oriented research; funded the development of online curricula on patient-oriented research that have reached thousands of stakeholders; developed a patient engagement resource center that has been accessed by tens of thousands of stakeholders; identified core competencies for research teams and research environments to ensure authentic and meaningful patient partnerships in health research; and shared these resources and learnings with stakeholders across Canada, North America, and internationally.

## Data Availability

Not Applicable.

## Abbreviations

SPOR: Strategy for patient-oriented research
PCORI: Patient-Centered Outcomes Research Institute
CIHR: Canadian Institutes of Health Research
OSSU: Ontario SPOR SUPPORT Unit
SUPPORT: Support for people and patient-oriented research and trials
WGTCD: Working Group for Training and Capacity Development
POR: Patient-oriented research
PERC: Patient Engagement Resource Centre
